# Current Advancements in the Diagnosis and Management of Mild-to-moderate Coronary Stenosis

**DOI:** 10.31083/RCM38822

**Published:** 2025-08-27

**Authors:** Cuimei Guo, Haizhu Gao, Xinxin Bian, Nan Lin, Lijun Gan, Xueying Chen

**Affiliations:** ^1^Colleague of Clinical Medicine, Jining Medical University, 272067 Jining, Shandong, China; ^2^Department of Cardiology, Shandong Key Laboratory for Diagnosis and Treatment of Cardiovascular Diseases, Jining Key Laboratory of Precise Therapeutic Research of Coronary Intervention, Affiliated Hospital of Jining Medical University, 272029 Jining, Shandong, China

**Keywords:** coronary artery disease, mild-to-moderate coronary artery stenosis, risk evaluation, intervention approaches

## Abstract

Coronary heart disease (CHD) is associated with increased morbidity and mortality. Acute cardiovascular events frequently occur in patients with coronary artery stenoses exceeding 70%. Although coronary revascularization can significantly improve ischemic symptoms, the inflection point for reducing mortality from CHD has yet to be reached. Therefore, the prevention and treatment of mild-to-moderate coronary artery stenosis should be given significant attention to more effectively reduce the incidence and mortality of acute events from CHD. Subsequently, a stenosis of less than 70% is used to characterize the incidence of mild to moderate coronary artery stenosis. While acute cardiovascular events caused by soft plaque and plaque rupture may not have a significant impact on hemodynamics, these events are detrimental and result in increased mortality. This review summarizes the methods available for detecting mild-to-moderate coronary artery stenoses, assessing risk, and understanding the mechanisms underlying adverse events. Moreover, this review proposes intervention strategies for preventing and treating mild to moderate coronary stenosis.

## 1. Introduction

The prevalence of coronary heart disease (CHD) in clinical practice is progressively increasing over time. It has emerged as one of the most significant threats to human health and mortality [1, 2]. The acute events of CHD are characterized by a stenosis of the vascular lumen and are commonly observed in patients with coronary artery stenoses exceeding 70%, necessitating interventions such as coronary stenting or coronary artery bypass graft surgery. Although these treatments significantly improve symptoms, they may not always reduce mortality rates for CHD. Therefore, emphasis should be placed on the prevention and treatment for CHD to reduce the incidence of acute events and mortality. This review focuses on patients with mild-to-moderate coronary artery stenoses, specifically those with coronary stenosis less than 70%. The timely identification and intervention in these patients can avert acute coronary events and reduce mortality.

Patients with mild-to-moderate coronary artery stenoses may not exhibit specific symptoms, however, it is associated with a significantly high prevalence rate in patients with CHD. According to a 2008 study involving 1000 asymptomatic middle-aged subjects undergoing coronary computed tomography angiography (CCTA), the incidence of coronary stenoses ranged from 25% to 74%, with an overall incidence of 8.3‰, while more than 75% exhibited severe stenosis. The incidence of coronary artery stenosis further escalates with progressive aging [3]. In 2019, a study conducted in South Korea on 601 healthy individuals who underwent CCTA revealed that 173 cases (28.8%) exhibited coronary artery stenosis, with an average stenosis rate of 25.8 ± 12.8% [4].

The presence of mild to moderate coronary artery stenosis may also contribute to the onset of acute cardiovascular events. The study included data from 42 patients who underwent invasive coronary angiography (ICA) before and after myocardial infarction (MI), and assessed the degree of stenosis of infarct-associated vessels in 29 patients with a new MI prior to the event. Among these patients, 19 (66%) had lumen diameters less than 50% and 28 (97%) had lumen diameters less than 70%. Interestingly, only a minority of MI (34%) were attributed to severe arterial occlusion observed on previous imaging studies, suggesting that atherosclerotic stenosis alone is not the primary etiology for cardiovascular events. Conversely, such events are more likely to occur in cases with mild-to-moderate stenosis [5]. Mild-to-moderate coronary artery stenosis may pose a greater risk compared to severe coronary artery stenosis due to the inherent instability of the plaque, making it more susceptible to rupture and thrombosis, thereby leading to vascular embolism and acute cardiovascular events. This study provides a comprehensive review on the detection, risk assessment, pathogenesis, and intervention strategies for mild to moderate coronary artery stenosis in order to increase attention to the prevention and treatment of patients with mild-to-moderate coronary artery stenosis.

## 2. The Definition of Mild-to-moderate Coronary Artery Stenosis

The definitions of mild to moderate coronary artery stenosis vary among different detection methods and institutions. In the CCTA test, the stenosis of each coronary artery is classified as follows: minimal (<30%), mild (30%–49%), moderate (50%–69%), or severe (≥70%) [6]. Coronary artery disease (CAD) is defined as none (0%), mild (1–49%), moderate (50–69%), or severe (≥70%) [7]. In 2022, the American Society of Cardiovascular Computed Tomography (SCCT) released a new reporting system to standardize CCTA reports. This system is called the Coronary Artery Disease Report and Data System (CAD-RADS). It ranges from CAD-RADS 0 indicating no atherosclerosis to CAD-RADS 5 indicating presence of at least one total vessel occlusion: 0% indicates no stenosis, 1%–24% indicates minimal stenosis, 25%–49% indicates mild stenosis, 50%–69% indicates moderate stenosis, 70%–99% indicates severe stenosis, and 100% indicates complete occlusion. It has been proven to be correlated with the degree of stenosis measured by ICA [8]. In general, mild-to-moderate coronary stenosis is generally defined as luminal stenosis less than 70%.

## 3. Method for Evaluating Mild-to-moderate Coronary Stenosis

The evaluation of patients with mild to moderate coronary stenosis involves two aspects. First, the assessment of the degree of lumenal stenosis and plaque structure is mainly conducted through ICA, CCTA, cardiac magnetic resonance (CMR), intravascular ultrasound (IVUS), optical coherence tomography (OCT), and near infrared spectroscopy (NIRS). Second, the functional evaluation focuses on the impact of hemodynamics in patients with coronary stenoses and includes fractional flow reserve (FFR), instantaneous wave-free ratio (iFR) and quantitative flow fraction (QFR).

### 3.1 Non-invasive Methods for CAD Assessment

#### 3.1.1 Coronary CT Angiography

CCTA is a non-invasive imaging modality that exhibits high sensitivity in detecting CAD. It can effectively visualize early coronary atherosclerosis in patients with specific conditions, including plaque load (calcium score, lesion segment points, etc.), plaque composition, and high-risk plaque based on anatomical and functional image information [9]. The use of enhanced CT measurements has been shown to predict cardiovascular events by assessing arterial calcium (CAC) scores [10]. The CAC score may be appropriate for specific asymptomatic patients with a moderate risk of CAD, enabling non-invasive evaluation of coronary atherosclerotic plaques and identification of high-risk plaques for risk stratification [11]. CCTA displays high-risk plaque characteristics (HRP), which include low density, positive remodeling, punctate calcification, and “napkin ring” appearance [12]. Risk models have demonstrated that the presence of high-risk plaque characteristics independently predicts cardiovascular events [13]. The calculation of the peripheral fat attenuation index (FAI) score on CCTA enables a direct quantification of the residual vascular inflammatory load [14].

A recent study has demonstrated that artificial intelligence-based novel CCTA assessment can rapidly and accurately identify and exclude stenoses, and are consistent with blinded, core laboratory-interpreted quantitative coronary angiography [15]. In comparison to ICA, CCTA tends to overestimate the degree of coronary artery diameter stenosis by 5.7%–8.5% during diastole (QCT-D) and by 9.4%–11.9% during systole (QCT-S) (*p* < 0.05). Therefore, it is recommended to diagnose the extent of stenosis based on QCT-D images [16]. Current research has established a positive correlation and consistent findings between CCTA and ICA [17]. Recent evidence from photon counting detector computed tomography (PCD-CT) suggests that this technique has more precise diagnostic performance for CAD [18]. CCTA also has certain disadvantages. The partial volume effect of the calcified plaque, severe coronary calcification, and heart rate can impact the assessment of the degree of coronary artery stenosis. Additional limitations include an increased risk of kidney injury with contrast media and the inability to open the stenoses in the patient’s blood vessels. However, due to continuous technological advancements, this imaging technique demonstrates high-resolution imaging capabilities, is non-invasive, is simple to perform, and is cost-effective. Consequently, it has made significant progress in enhancing diagnostic performance and reducing radiation exposure. As a result, it is widely utilized for early screening and diagnosis of cardiovascular diseases and has become a reliable diagnostic tool for both elective and emergency care [9, 19]. These attributes contribute to a higher inclination among patients with mild-to-moderate coronary stenosis to select CCTA.

#### 3.1.2 Cardiac Magnetic Resonance Angiography (CMRA)

CMR is a highly effective noninvasive imaging technique for evaluating coronary vascular stenoses, that can comprehensively assess the heart’s structure, function, blood perfusion, and characteristics. It provides detailed imaging features of both the vascular lumen and wall and is non-invasive and has no radiation exposure. CMR offers high spatial and temporal resolution, excellent reproducibility and accuracy, even in individuals with a high body mass index [20]. A multicenter trial conducted by Kato *et al*. [21] demonstrated that 1.5-T whole-heart CMRA exhibits a high negative predictive value of 88%. This indicates that whole-heart CMRA is effective in excluding the presence of coronary artery disease and thus plays a valuable role in reducing the need for invasive angiography, as opposed to the technical limitations of CCTA, which results in false positive or negative results in the presence of severe calcification [22]. CMRA has better diagnostic performance than CCTA in detecting significant stenosis of coronary artery segments with moderate to severe calcified plaques [23]. Kim *et al*. [24] demonstrated that CMR can accurately identify an increase in coronary artery wall thickness in patients with non-significant (10%–50%) CAD, while maintaining lumen size. This finding contributes to the enhancement of risk stratification for individual patients, facilitating early treatment and prevention of acute coronary events. The sensitivity of CMR is relatively high, but the long imaging duration and high cost impose a burden on patients. In addition to concerns regarding gadolinium contrast media, CMR cannot be performed in patients with non-MR pacemakers or implantable cardioverter defibrillators, significantly limiting its clinical application [25]. In addition, CMRA is subject to motion artifacts due to both respiration and cardiac pulsation, which significantly limit its image quality. It’s lower spatial resolution hinders the assessment of small coronary vessels.

### 3.2 Invasive Methods for CAD Assessment

#### 3.2.1 Invasive Coronary Angiography

ICA remains the “gold standard” for the diagnosis of coronary artery stenosis. It can be used to make a definitive diagnosis of the location, extent, and severity of the lesion, and the condition of the vessel wall, to determine the treatment plan (interventional, surgical, or medical management), and can also be used to determine the efficacy of the treatment. However, its methodology for determining the degree of coronary stenosis based on the degree of contrast filling the vessel does not allow for assessment of either the specifics of the lumen or the characteristics and morphology of the diseased plaque. The invasive nature and cost of the technique limit its usefulness in assessing plaque stability and predicting the development of acute coronary syndromes [26]. The utilization of this method for assessing patients with mild-to-moderate coronary artery stenoses is subject to certain limitations, which include potential complications, increased cost, as well as radiation exposure and discomfort experienced by both patients and operators. Additionally, there exists inter-operator variability in assessing coronary stenosis definitions which may impact its clinical application in patients with mild-to-moderate stenoses.

#### 3.2.2 Invasive Anatomical Assessment

In addition to assessing the degree of stenosis of the vessel lumen, detailed evaluation of the plaque structure is more comprehensive and meaningful in predicting its prognosis. IVUS, OCT, NIRS are utilized to identify high-risk plaques and assess plaque inflammation. IVUS is one of the most widely used invasive imaging methods for studying coronary plaques. Radiofrequency ultrasound echo data identify plaque components, including plaque burden, expansive remodeling, necrotic core, calcification, and neovascularization [27]. In addition, despite the inability to measure the thickness of the thin fibrous cap, a prospective study has shown that IVUS-defined thin-cap fibrous atherosclerotic tumors (TCFA) are associated with major future adverse cardiovascular events [28]. OCT is the second major invasive imaging method for coronary artery plaques. OCT uses near-infrared light with a wavelength of 1.3 µm to image the plaques, and the resolution can reach up to 20 µm. It can clearly show the thickness of the fibrous cap and the collagen content, effectively identify macrophages and neovascularization, and accurately determine plaque rupture, and the presence of thrombi [29]. However, due to the high scattering effect of its imaging medium, OCT is limited by longer image acquisition times and numerous artifacts. It is difficult to evaluate the deep plaque structure, and its ability to distinguish between calcified areas, such as microcalcifications with a diameter of less than 5 µm, and the lipid core, is also relatively poor [30]. To address this challenge, a new form of OCT with 1 to 2 µm resolution, termed micro-OCT (µOCT), has been developed. This technology can visualize key events in the development and progression of atherosclerosis at the cellular and molecular levels, including leukocyte extravasation, fibrin filament formation, extracellular matrix (ECM) production, endothelial denudation, as well as microcalcification, cholesterol crystal formation, and fibrous cap penetration [31]. High-resolution OCT excels in detecting potential palque vulnerability, such as thin fibrous caps in atherosclerotic plaques. The most reliable imaging method is confirming plaque erosion. NIRS utilizes the characteristic emission spectra generated by the interaction of plaque content with photons and is suitable for assessing the lipid content of plaques, especially in the case of positive remodeling, large, deep, and lipid-rich necrotic cores (maximal lipid core burden index in a 4 mm segment ≥400) [32, 33]. The invasiveness and high cost of these tests restrict their clinical utility in patients with mild to moderate arterial stenosis. Nevertheless, for symptomatic patients with mild-to-moderate narrowing, these tests can evaluate treatment options. The selection of appropriate methods for coronary artery assessment can be based on the individual’s detection objectives and conditions, particularly for patients with mild-to-moderate coronary artery stenoses.

### 3.3 Invasive Functional Assessment

FFR is the most effective indicator for assessing the hemodynamic significance of a coronary artery stenosis. FFR represents the ratio of blood flow through a stenosed coronary artery to that in the absence of stenosis, derived from applying Poiseuille’s law and calculating the ratio of arterial pressure distal to proximal to the stenosis. FFR serves as the “gold standard” for functional evaluation at ICA [34]. The application of computational fluid dynamics in estimating FFR based on CCTA studies has demonstrated a strong correlation with invasive measurements, as evidenced by preliminary research findings [35]. In evaluating percutaneous coronary intervention (PCI) outcomes in patients with moderate stenosis (40%–70%), FFR guidance is non-inferior to IVUS guidance regarding composite endpoints such as death, myocardial infarction, or revascularization at 24 months [36]. An FFR ≤0.80 or lower is recommended as a diagnostic threshold for significant coronary artery disease [37]. In patients with moderate stenosis, FFR_CT_ strategies appear to be associated with lower ICA, ICA without obstructive disease, and 1-year major adverse cardiac events (MACE) compared to anatomic CCTA strategies alone. These findings suggest that the assessment of hemodynamic significance holds greater predictive value for prognosis than for purely anatomical considerations [38]. In addition, it would be beneficial to also emphasize the importance of post-PCI functional assessment, as evidence suggests that achieving optimal post-PCI functional status is associated with better clinical outcomes compared to procedures guided solely by pre-PCI FFR [39]. Recent research has introduced a novel tool, the pressure pullback gradient (PPG) index, which assesses not only the severity of stenosis but also the pattern of disease distribution (focal vs. diffuse) [40]. The combination of FFR and PPG may offer additional insights for identifying high-risk stenoses [41]. In addition, iFR and QFR can effectively determine the correlation between coronary artery stenosis and hemodynamics. A large number of studies have shown that iFR, QFR, and FFR show high correlation and consistency in evaluating interventional results, and have obvious technical advantages in the evaluation of patients with moderate coronary artery stenoses [42, 43, 44].

## 4. Risk Assessment for Patients With Mild-to-moderate Coronary Stenosis

The presence of mild-to-moderate coronary artery stenosis did not exert a significant impact on hemodynamics. Nevertheless, owing to its high prevalence, the incidence rate of cardiovascular events remains elevated in this population. Given its widespread occurrence, the absolute number of incidents within this specific group remains substantial [45].

In comparison to patients without coronary artery disease, those with <50% luminal stenosis have a threefold increased risk of MACE [46]. Among patients undergoing CCTA examination for suspected CHD, approximately 23.1% exhibited early coronary atherosclerotic lesions, while the incidence of MACE during the 3-year follow-up was estimated to be around 3.7% [47]. A study by Yeonyee *et al*. [48] involved 207 patients suspected of having CAD. CMRA assessed the presence of significant coronary stenosis (≥50%) subgroups, and Kaplan-Meier curves showed significant differences in annual event rates (6.3% and 0.3%; *p* < 0.001) for all cardiac events during a median follow-up of 25 months. A study by Yorgun *et al*. [49] included a cohort of 1115 patients diagnosed with mild-to-moderate coronary stenosis, with a median follow-up of 29.7 months, during which a total of 29 end-point events occurred. In a multicenter, multinational study involving 15,187 patients, the annual incidence rate of MACE in patients with nonobstructive (<50%) CAD was found to be 1.22% during a mean follow-up of 2.4 ± 1.2 years [7]. A study by Chow *et al*. [50] included a total of 27,125 consecutive patients who underwent CCTA. The results showed that the presence and extent of nonobstructive (<50%) CAD predicted mortality, with a 6% (95% CI: 1%–12%, *p* = 0.021) increase in the risk of death for each additional nonobstructive plaque. In 2016, a study conducted in Australia enrolled 1469 patients diagnosed with low and moderate risk coronary heart disease. Over a follow-up period of 7.8 years, no MACE were observed in patients without any detectable coronary artery stenosis as monitored by CCTA. The incidence of MACE was found to be 1.3% in patients with a stenosis less than 10%, while it increased to 2.5% in those with a stenosis less than 50%. Notably, the incidence of MACE rose to 3% among patients with stenoses ranging from 50% to 70% [51]. The same group released data in 2020, providing a follow-up period of approximately 10 years [12]. The findings revealed that the incidence of MACE was as follows: 0.2% in patients without coronary stenosis, 2.8% in patients with stenosis less than 10%, 3.5% in patients with stenosis less than 50%, and 5.7% in patients with stenosis ranging from 50% to 70%. Taron *et al*. [13] conducted a CCTA examination on 4686 patients, among whom 33.8% and 13.0% exhibited stenosis degrees of 30%–49% and 50%–69%, respectively. During the median follow-up of 26 months, there were a total of 86 primary endpoint events, including 42 deaths, 17 MI, and 27 unstable angina events. The study demonstrated that mild/moderate stenosis (HR: 1.91; *p* = 0.011) and the presence of ≥2 HRP features (HR: 2.40; *p* = 0.008) independently predicted adverse events in this population. The Evaluation of Ischemic Syndrome in Women revealed a 10-year mortality rate of 20% among women referred for coronary angiography due to ischemic signs and symptoms, with one-third of fatalities occurring in women without obstructive CAD [52]. A 9-year follow-up of 2522 middle-aged and elderly patients with nonobstructive CAD in China in 2022 showed a mortality rate of 4.35% in patients with <50% luminal stenosis, a 7-fold increase in risk compared with patients without any CAD [53]. In summary, the incidence of MACE varies across different populations, exhibiting an overall upward trend over time.

A summary of these studies involving patients with mild-to-moderate coronary stenosis is presented in Table 1 (Ref. [7, 12, 13, 48, 49, 50, 51, 53]).

**Table 1.** **Research on cardiovascular events in patients with mild-to-moderate coronary artery stenosis**.

	Time	Country	Inclusion criteria	Subjects	Follow-up time (median)	Incidence and outcome events
Yeonyee *et al*. [48]	2012	Japan	Suspected CAD	207	25 months	The degree of stenosis <50% of patients all heart attacks by 0.3%
Yorgun *et al*. [49]	2013	Türkiye	Mild-to-moderate coronary artery stenosis (<70%)	1115	29.7 ± 13.2 months	The incidence of MACE was 2.6%
Nakazato *et al*. [7]	2014	Multiple centers	Nonobstructive CAD	15,187	2.4 ± 1.2 years	The incidence of MACE was 1.22% in patients with stenosis <50%
Chow *et al*. [50]	2015	Multiple centers	Normal or non-obstructive CAD (1%–49%)	27,127	27.2 months	Mortality rate of 0.44%
Feuchtner *et al*. [51]	2017	Australia	CAD of low to moderate risk	1469	7.8 years	The incidence of MACE was 1.3%, 2.5%, and 3% in patients with stenosis <10%, <50%, and 50–70%, respectively
Senoner *et al*. [12]	2020	Australia	CAD of low to moderate risk	1469	10 years	The incidence of MACE was 2.8%, 3.5%, and 5.7% in patients with stenosis <10%, <50%, and 50–70%, respectively
Taron *et al*. [13]	2021	Multiple centers	Left main stenosis (1%–49%) or other (1%–69%)	2890	26 months	The incidence of endpoint event was 3.3%
Huang *et al*. [53]	2024	China	Nonobstructive CAD	2522	9 years	Annualized all-cause mortality: 4.9%, 8.2%, 13%, and 18% in the no CAD, 1-, 2-, and 3-vessel nonobstructive CAD groups, respectively

Annotation: CAD, coronary artery disease; MACE, major adverse cardiovascular events.

## 5. Mechanisms of Adverse Cardiovascular Events in Mild-to-moderate Coronary Stenosis

**Fig. 1.**
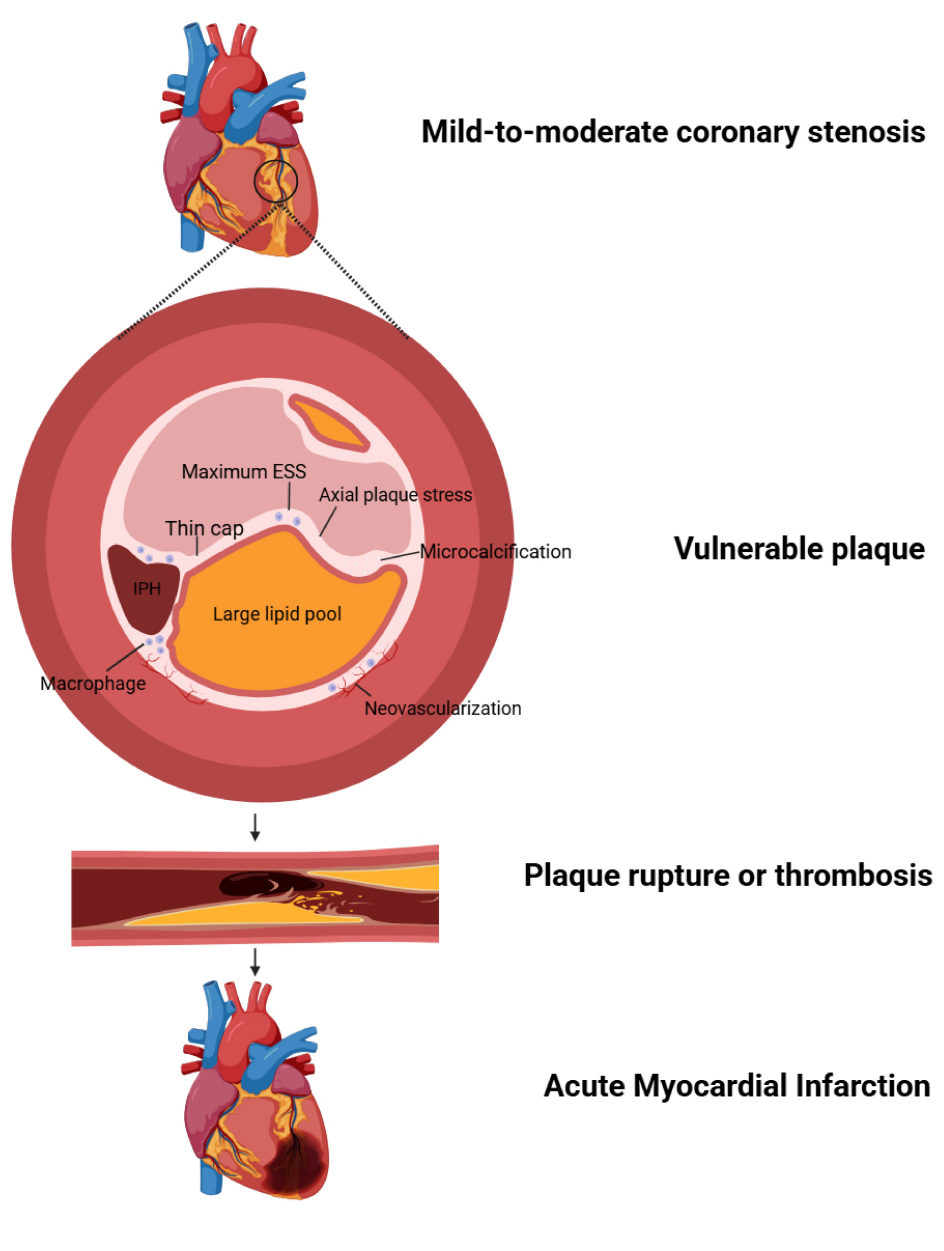
**The pathophysiological mechanisms underlying acute cardiovascular events in patients with mild-to-moderate coronary stenosis**. ESS, endothelial shear stress; IPH, intraplaque hemorrhage. Created with Biorender.com

The primary etiological mechanism leading to major adverse cardiovascular events is the development of focal necrosis in the corresponding myocardium due to persistent ischemia caused by severe obstruction of the coronary lumen. In patients with mild-to-moderate coronary stenosis, the occurrence of cardiovascular events, in addition to the classical pathogenesis of the natural progression of coronary atherosclerosis, is closely related to the degree of plaque burden throughout the coronary arteries (Fig. 1), including both anatomical and biomechanical characteristics [54].

### 5.1 Plaque Anatomical Features

The findings from autopsy studies have revealed that coronary luminal thrombosis can be attributed to three distinct morphological entities: plaque rupture (55–65%), plaque erosion (30–35%), and calcified nodules (2–7%) [55]. Plaque rupture of the precursor lesion, known as “vulnerable plaque”, is considered the primary mechanism for sudden lumen thrombosis in patients with mild-to-moderate coronary stenosis, leading to adverse cardiac events [13, 56]. Studies have demonstrated that more susceptible ruptured plaques are obstructive plaques characterized by a thin fibrous cap (<65 microns), an abundant lipid-rich necrotic core, low remodeling index, microcalcifications, along with infiltration of inflammatory cells, apoptosis, lipids, new blood vessels and intraplaque neovasculature. This type of plaque is often referred to as TCFA [57, 58]. Despite the absence of ischemia symptoms at baseline assessment, patients with TCFA had a five-fold higher rate of MACE during an 18-month follow-up period compared to those without TCFA (>80% accounted for future MACE) [59]. Furthermore, positive TCFA status after a 5-year follow-up was associated with an increased risk of long-term adverse events [60]. In recent years, a distinct mechanism known as plaque erosion (PE) has gained recognition and attention. It is characterized by an intact fibrous cap and thrombus formation at the site of a de-endothelialized atherosclerotic plaque, including increased smooth muscle cells, neovascularization, and the potential role of hyaluronic acid as a marker of inflammation and endothelial desquamation. Studies have shown that PE is associated with acute coronary syndrome (ACS) in at least one third of patients, especially non-ST-segment elevation ACS [41, 61, 62]. Calcified nodules, although uncommon, are of great clinical significance. It results in uneven stress on the plaque fibrous cap, greatly increasing plaque vulnerability and significantly increasing the risk of acute coronary syndromes [58].

### 5.2 Alteration in Plaque Composition

Macrophages, are the primary inflammatory cells in plaques and play a crucial role in plaque vulnerability. During the process of phagocytosing cholesterol crystals, macrophages contribute to ROS cluster-mediated cellular damage and plaque rupture by releasing TNF-α, interleukin (IL)-1/6, platelet-derived growth factor (PDGF), TGF-α/β, and other cytokines [63, 64]. The migration ability of macrophage-derived foam cells is diminished due to cholesterol load, leading to the activation of pro-inflammatory cytokines and chemokines. This results in secondary necrosis and release of cell components into the plaque environment, promoting the formation of a lipid-rich core and increasing plaque vulnerability [63]. The overproduction of matrix metalloproteinase (MMP) with interstitial collagenase activity also occurs. MMP-9 can degrade the ECM and regulate chemokines, thin the fiber cap, increase the size of atherosclerotic plaques, promote monocyte/macrophage infiltration, increase inflammation, enhance plaque vulnerability, and eventually lead to plaque rupture [65, 66]. The MMP-8 enzyme facilitates the migration of endothelial cells by degrading the surrounding ECM, thereby releasing a range of angiogenesis promoters and inhibitors from the ECM to enhance plaque-associated angiogenesis [67].

The new blood vessels typically consist of a single-layer ECM on the basement membrane, lacking support from smooth muscle cells (SMC). This renders the immature plaque blood vessels highly susceptible to damage. Activation of macrophages and mast cells can result in the release of matrix metalloproteinases and inflammatory cytokines, further contributing to the impairment of these newly formed blood vessels [68]. New blood vessels enhance macrophage infiltration and promote red blood cell sources of cholesterol in the lipid core transport, thereby accelerating the progress of atherosclerosis and intraplaque hemorrhage (IPH), resulting in an increase in plaque volume, and promoting plaque rupture [69]. In contrast, Brezinski *et al*. [70] believes that angiogenesis is a supply line for repairing cells in the healing area of the coronary artery. In vulnerable plaques with long necrotic cores, supply lines to the damaged area may not be reliably established, leading to plaque instability [70].

The involvement of mast cells is also crucial in atherosclerotic plaque rupture. The fibrous cap consists of vascular smooth muscle cells (VSMCs) and their collagen production, rendering the thin cap susceptible to rupture. The presence of activated mast cells is elevated in ruptured coronary plaques, leading to the secretion of heparan proteoglycans that effectively inhibit VSMC proliferation and reduce their collagen-producing capacity. The Mi enzyme, a neutral serine protease secreted by VSMCs, plays a role in inhibiting collagen synthesis mediated by SMC through its dependence on transforming growth factor beta and activates MMP-1 for extracellular matrix degradation. Furthermore, Mi enzyme promotes SMC apoptosis by degrading fibronectin, an essential component for SMC adhesion to the extracellular matrix. Both mechanisms contribute to weakening and eventual rupture of the thin fibrous cap in atherosclerotic plaques [71, 72]. The presence of a large necrotic core rich in lipid and collagen, along with the absence of cells and the eccentric distribution of the lipid core caused by patches of circumferential stress and shoulder area rearrangement, leads to an increased vulnerability of plaques [73].

Vascular calcification is associated with an elevated risk of cardiovascular disease. Lineage tracing studies in mice have shown that 98% of plaque bone cartilage cells originate from VSMC, which are formed in response to high levels of calcium and phosphate, contributing to bone cartilage formation phenotypes [74]. Microcalcification (<50 microns) is linked to increased inflammation and significantly increases tissue stress within the fibrous cap. When this stress exceeds the threshold for cap failure, it leads to plaque rupture, thrombosis, and vascular occlusion [75]. Microcalcification plays a crucial role in destabilizing atherosclerotic plaques. Even supposedly stable plaques become vulnerable to rupture when microcalcifications are introduced into the fibrous cap; however, these microcalcifications must also be located in areas where background tissue stress is sufficiently high to promote plaque rupture [58].

### 5.3 Biomechanical Properties

In addition to the properties and components of the plaque, coronary biomechanical forces also play a crucial role in the progression of coronary plaques, including endothelial shear stress (ESS), plaque structural stress (PSS), and axial plaque stress (APS) [76]. Wall shear stress (WSS) refers to the parallel friction force exerted by blood flow on the endothelial surface, which is considered as the key hemodynamic force influencing the occurrence, development, and transformation of atherosclerotic plaques. Low WSS disrupts the homeostatic atherogenic protective properties of normal endothelium, resulting in blood flow stagnation in that region and promoting plaque formation. High WSS can induce damage to vascular endothelial cells through increased expression of matrix-degrading metalloproteinases, leading to inflammation and oxidative stress. This ultimately increases plaque vulnerability and raises the risk for thrombosis [77].

PSS represents mechanical stresses located within an atherosclerotic plaque or arterial wall caused by changes in arterial pressure and cardiac motion-induced vasodilation, and stretching. PSS significantly increases with fibrous cap thickness reduction, necrotic core area enlargement, and microcalcification accumulation. Elevated PSS promotes macrophage accumulation while limiting smooth muscle cell activity through activated matrix metalloproteinase expression. These processes lead to matrix degradation and thinning fibrous caps until PSS exceeds their mechanical strength causing rupture. APS refers to the stress exerted on the surface of atherosclerotic plaque in the vessel wall, which is responsible for force imbalance within the lesion. Plaque formation and growth alter the mechanical properties of the vessel wall, resulting in localized areas of stress. Elevated APS increases the likelihood of plaque rupture and detachment, leading to adverse cardiovascular events. Furthermore, a significant inverse correlation was observed between APS and lesion length, which explains why short lesions and focal lesions have a higher incidence of plaque rupture compared to diffuse lesions [78].

## 6. Intervention Strategies for Patients Presenting With Mild-to-moderate Coronary Artery Stenosis

### 6.1 General Intervention

The general intervention in patients with mild-to-moderate coronary stenosis follows the same overall strategy for preventing coronary atherosclerotic stenosis: maintaining a healthy diet and normal body weight, engaging in regular exercise, quitting smoking, and regularly monitoring blood pressure, cholesterol levels, and blood glucose. Additionally, incorporating rehabilitation training into the treatment of patients with mild-to-moderate coronary stenosis has shown significant effects by reducing the incidence of vulnerable plaques and improving plaque composition, thereby lowering the risk of cardiovascular disease [79]. Furthermore, a study has indicated that certain traditional Chinese medicine treatments such as acupuncture may serve as effective adjunct therapies for patients with stable angina pectoris and moderate (40%–70%) CAD by regulating brain activity [80].

### 6.2 Drug Treatment

Selective drugs target the modification of the atherosclerotic plaque, address risk factors, and manage the environment of clots and inflammation. These drugs include statins and antiplatelet agents.

#### 6.2.1 Lipid-lowering Drugs

The administration of statins has been shown to have lipid-lowering properties, improve endothelial function, exhibit antioxidative and anti-inflammatory effects, possess antithrombotic properties, and effectively prevent the development of atherosclerosis. Furthermore, they promote plaque stability and reduce the incidence of cardiovascular disease and mortality [50, 81]. Statin treatment has been shown to effectively reduce the progression of low attenuation plaques and non-calcified plaques [82]. A 2013 review [83] retrospectively analyzed a cohort of 1952 patients with coronary stenosis ranging from 1–69% on CCTA. Statins were found to have significant benefits in patients with non-calcified or mixed plaques (HR: 0.47, *p* = 0.047). However, no benefit was observed in reducing adverse events. In patients with stable angina pectoris and mild-to-moderate coronary artery stenosis, a daily dose of 40 mg atorvastatin is superior to 20 mg [84]. High-intensity statin therapy results in a significant increase in the minimum thickness of fibrous cap and reduction in the presence of vulnerable plaques, potentially transforming up to three-quarters of plaques into a more stable phenotype within 13 months after infarction-related coronary artery events [85]. However, it is important to note that they may also increase the incidence of liver and kidney damage, myalgia, rhabdomyolysis, among other adverse effects. Therefore, further investigation is required to assess the benefits and risks specifically in patients with mild-to-moderate coronary artery stenosis that are not hemodynamically significant.

The Proprotein convertase subtilisin/kexin 9 (PCSK9) inhibitors decrease the degradation of the low-density lipoprotein (LDL) receptors. They are primarily used to lower low-density lipoprotein cholesterol (LDL-C) and are particularly useful in patients with pure familial hypercholesterolemia. In addition, studies have shown that PCSK9 inhibitors decrease the degradation of the LDL receptors [86]. A study has shown that the use of PCSK9 inhibitors is accompanied by a beneficial remodeling of the inflammatory load in patients with atherosclerosis. This effect may be, or is partially, independent of their ability to lower LDL-C and may provide additional cardiovascular benefits [87]. The 2024 edition of the European Society of Cardiology (ESC) guidelines recommends that all patients with chronic coronary syndrome (CCS) should be treated with the highest tolerated dose of a high-intensity statin in order to achieve an LDL-C <1.4 mmol/L and a ≥50% reduction from baseline. PCSK9 inhibitors are recommended for patients who do not achieve the goal after treatment with the highest tolerated dose of statin combined with ezetimibe. In addition, a recommendation for bempedoic acid as third-line therapy was added [88].

#### 6.2.2 Antiplatelet Drugs

Aspirin is the most classical antiplatelet drug, which inhibits platelet aggregation and activation induced by neutrophils. It also reduces nitric oxide production by limiting endothelial prostacyclin synthesis and protects low-density lipoproteins from oxidative modification, thereby impeding the progression of atherosclerosis [89]. A multicenter study conducted in seven countries demonstrated that aspirin treatment did not significantly reduce the risk of major cardiovascular events in patients with moderate risk [90]. In a study involving patients with non-obstructive stenosis (1%–49%), the use of aspirin did not have a significant impact on MACE, baseline all-cause mortality, or MI [91]. The findings of a meta-analysis demonstrated that aspirin exhibited a significant reduction in the incidence of cardiovascular events among individuals without cardiovascular disease. However, it was associated with an elevated risk of major bleeding and did not demonstrate any impact on mortality [92]. This limits its use in individuals with low risk or those who are intolerant to aspirin.

In 2019, Lee *et al*. [93] enrolled 100 diabetic subjects with mild-to-moderate coronary stenosis as assessed by CCTA. The findings revealed that treatment with ciliostazole for a duration of 12 months resulted in significant reductions in coronary artery stenosis and non-calcified plaque components, along with an increase in high density cholesterol levels, and decreased levels of triglycerides, liver enzymes, and highly sensitive C-reactive protein. Furthermore, it was observed that abdominal visceral fat area and insulin resistance were also reduced as a result of this intervention. In a subsequent extended study with a median follow-up period of 5.2 years [94], cilostazole treatment demonstrated its superiority over aspirin in reducing the incidence of cardiovascular events among subclinical CAD patients with diabetes.

#### 6.2.3 Other Drugs

A study demonstrated that the administration of isosorbide mononitrate combined with nicorandil can effectively reduce levels of inflammatory factors, thereby improving myocardial ischemia and alleviating symptoms associated with mild-to-moderate coronary stenosis and unstable angina [95]. Recent studies have demonstrated that sodium-glucose transporter 2 inhibitors (SGLT2-I) exhibits a beneficial effect on improving lipid deposition, inflammation, and thickness of atherosclerotic plaques, while also reducing MACE by half—the most favorable clinical outcome—in diabetic patients with multi-vessel nonobstructive coronary artery stenosis (20–49%). Furthermore, the implementation of ICA and OCT following SGLT2-I treatment has shown to predict a 65% lower risk of MACE during one-year follow-up [96]. The anti-inflammatory effects of colchicine are distinct, and in a randomized trial involving patients with chronic CAD, those who received a daily dose of 0.5 mg of colchicine exhibited a significantly reduced risk of cardiovascular events compared to the placebo group [97]. Despite numerous reports on drug intervention in patients with mild-to-moderate coronary artery stenosis, the specific mechanism and application criteria remain unclear, necessitating the need for further extensive clinical trials and studies to confirm these findings.

### 6.3 Percutaneous Coronary Intervention

FFR is employed to assess whether a stenosis leads to ischemia, enabling an evaluation of moderate coronary artery lesions suitable for PCI [98]. An international multicenter prospective study in Europe and Asia demonstrated that the risk of cardiac death or MI associated with moderate coronary artery stenosis based on FFR ≥0.75 was <1% per year and was not reduced by PCI, and that performing PCI did not improve prognosis or anginal status, nor did it reduce the use of antianginal medications. However, in patients with significant functional stenosis (FFR <0.75), PCI can significantly improve the functional class (CCS class is the Canadian Cardiovascular Society’s rating scale for exertional angina) [99, 100]. A 2014 study enrolling 1220 patients with stable coronary artery disease who had at least one stenosis and an FFR ≤0.80 were randomly assigned to receive either FFR-guided PCI plus medication or medication alone. It found that the incidence of the primary endpoint was significantly lower in the PCI group than in the medication group (8.1% vs. 19.5%, risk ratio 0.39; 95% CI: 0.26–0.57; *p* < 0.001) [98]. In summary, for patients with mild-to-moderate coronary stenosis, the criterion for hemodynamic reconstruction is an FFR value of 0.80 or lower (indicating a ≥20% reduction in coronary blood flow). Although hemodynamic significance holds greater prognostic value than anatomical significance, FFR-guided revascularization may emerge as a popular direction for future clinical care in patients with mild-to-moderate coronary stenosis. A study has shown that the FFR positivity rate (FFR <0.8) can be as high as 54% in patients with coronary artery stenosis of less than 50% [101]. In addition, numerous studies have shown that even in the presence of a negative FFR, high-risk plaque characteristics are still risk factors for adverse cardiovascular events [59, 60, 102]. The recently published PREVENT trial (NCT02316886) focused on a cohort of patients with coronary artery stenosis >50% (mean diameter stenosis 54.5%) and the presence of nonflow-restrictive lesions (FFR >0.80) but having characteristics associated with a high-risk for plaque instability. The results showed that, compared with optimal medical therapy alone, PCI can significantly reduce the incidence of MACE caused by high-risk vulnerable plaques [103]. However, caution should be exercised when considering implanting stents or stenting non-ischemic plaques with mild-to-moderate coronary stenosis due to the potential risk of developing new stent-related complications such as in-stent thrombosis or in-stent restenosis. Recent studies have indicated that drug-coated balloon (DCB) therapy can reduce plaque burden and even increase fibrous cap thickness [104, 105]. Nevertheless, there is currently insufficient substantial evidence regarding the safety and efficacy of DCB in treating patients with mild-to-moderate coronary artery stenosis.

## 7. Conclusion

Although patients with mild-to-moderate coronary artery stenosis may not exhibit characteristic clinical symptoms, they have a high incidence and are at a high risk for cardiovascular events. Therefore, early intervention to reduce these events is of great significance in the management of CHD. Currently, there is a lack of specific risk assessment models for patients with mild-to-moderate arterial stenosis, and the benefits and risks associated with PCI and drug interventions are still under debate. Clear guidelines for control are also lacking. Thus, further research is needed to investigate the unique mechanisms underlying mild-to-moderate arterial stenosis and develop effective intervention strategies aimed at reducing both the progression of coronary artery narrowing and acute events as well as reducing mortality rates associated with CHD.
